# Retraction Note: New insight of the efficacy trimetazidine in patients with peripheral arterial disease: a meta-analysis

**DOI:** 10.1186/s43044-024-00496-0

**Published:** 2024-05-17

**Authors:** Ketut Angga Aditya Putra Pramana, Ni Gusti Ayu Made Sintya Dwi Cahyani, Yusra Pintaningrum, Basuki Rahmat

**Affiliations:** 1 General Practitioner of Mataram University, Mataram, Indonesia; 2 Interventional Cardiology Division, Cardiology and Vascular Department, Faculty of Medicine, Mataram University, Mataram, Indonesia

**Retraction Note to: The Egyptian Heart Journal (2024) 76:31** 10.1186/s43044-024-00461-x

The authors have retracted this article because they do not have ownership of the data presented. All authors agree with this retraction.

